# Identification of Demographic Variables Influencing Dementia Literacy and Risk Perception Through a Global Survey

**DOI:** 10.3389/fpubh.2021.660600

**Published:** 2021-06-08

**Authors:** Becky R. Horst, Joyla A. Furlano, Michelle Y. S. Wong, Sabrina D. Ford, Brenna B. Han, Lindsay S. Nagamatsu

**Affiliations:** ^1^Exercise Mobility and Brain Health Lab, Department of Neuroscience, Western University, London, ON, Canada; ^2^Exercise Mobility and Brain Health Lab, School of Kinesiology, Western University, London, ON, Canada

**Keywords:** dementia literacy, risk factors, demographics, public health, global

## Abstract

Dementia literacy is important for risk mitigation and preventative strategies before disease onset. The aim of our study was to investigate dementia literacy and how demographic characteristics influence these perceptions in order to provide evidence for how dementia-centered public health initiatives should structure their focus. We conducted a globally administered online survey, through Amazon Mechanical Turk (mTurk). Survey items evaluated: (1) personal perception on the preventability of dementia, and (2) risk awareness of lifestyle factors. Differences in risk scoring between the 598 respondents were compared using Kruskal-Wallis testing factored by demographic categorizations. Most of the sample demonstrated understanding that lifestyle factors contribute some risk toward dementia, though these risk scores were generally low. Differences in risk scoring varied by demographic characteristics. Women, older adults, those with non-post-secondary attainment, below average income, and White background tended to report lower risk scores. Public health education and initiatives for dementia prevention should focus on lifestyle risk factors, in addition to considering the barriers related to the demographic factors identified that may prevent populations from accessing programs and information.

## Introduction

Nearly 50 million people worldwide are living with some form of dementia, with 10 million new cases occurring each year (1). Despite the widespread impact of dementia, public awareness surrounding dementia continues to demonstrate large gaps, especially for public knowledge concerning the prevention and risk reduction of dementia. A recent systematic review indicated that nearly 50% of the general population, from various countries surveyed, viewed dementia as a normal part of aging and a non-preventable part of living (2). Similarly, in the Netherlands (3) and United States (4) individuals demonstrated difficulty in identifying risk factors related to dementia. Despite the general populations' beliefs, dementia is not a normal part of aging and certain factors have been linked to increasing dementia risk. It is evident that dementia literacy in the general population continues to be a critical area for improvement for public health education.

Dementia literacy, one's beliefs regarding dementia and application of beliefs, is an important public health concept for risk mitigation and preventative strategies before disease onset (5). The improvement of dementia literacy can aid individuals in both identifying risk factors related to dementia, but also increase recognition that many risk factors are also modifiable. Improving dementia literacy for modifiable risk factors, such as those related to lifestyle (e.g., physical activity, diet, mental health) can be a critical tool in encouraging individuals to change their behaviors in order to decrease the risk of dementia or slow disease progression (6, 7).

Evaluations of dementia beliefs in the general population have begun to identify where these literacy shortfalls lie. However, dementia beliefs may be influenced by a variety of factors such as education, ethnicity, income, and sex. Identifying where the general public's dementia beliefs falters, and how demographic characteristics further influence these opinions, can better inform the content and audience that public health initiatives should target for improved dementia literacy, especially regarding modifiable risk factors.

The aim of our study was to investigate perceptions surrounding risk factors and beliefs related to dementia. Specifically, we aimed to identify the underlying demographics that influence these perceptions in order to provide evidence for how dementia-centered public health initiatives should structure their focus in order to improve dementia literacy. Therefore, we conducted a globally administered online survey in order to evaluate these perceptions on a large scale across varying demographic profiles.

## Methods

An online survey was conducted through Amazon Mechanical Turk (mTurk), allowing English-speaking mTurk users aged 18 years and older to participate. The use of mTurk has been shown to be a valid platform for collecting diverse demographic respondents (8, 9) and reliable data compared to lab-based studies (10). Prior to the survey, users were provided with an online letter of information and consent. Those that consented were directed to the questionnaire hosted via the online survey software Qualtrics. All procedures of this study were approved by the Non-Medical Research Ethics Board at Western University, ID #113040.

Questions to assess dementia literacy were developed based on information from the Alzheimer Society of Canada website (11) and previous dementia literacy surveys (12, 13). Survey items evaluated: (1) personal perception on the preventability of dementia, and (2) risk awareness of lifestyle factors that have the potential to increase dementia risk. Participants were asked to rate, on a scale of 0 to 100%, their perception of dementia preventability based on the statement, “to what extent do you believe that Alzheimer's disease and dementia are preventable?.” Additionally, each potential risk factor was individually scored on a seven-point scale for its possibility of increasing dementia risk. Values ranged from one, representing “no risk,” to seven, representing “high risk.” We included 14 risk factors for evaluation: 11 of which are strongly supported as factors for increasing dementia risk (old age, genetics, physical inactivity, unhealthy diet, stress, heart disease, head injury, mental illness, high alcohol consumption, smoking, and social isolation). In addition, we included two factors that have limited supported associations to dementia risk (exposure to toxins, and viral/bacterial infections), and one non-empirical factor (God's will).

Socio-demographic variables of sex, age, education, and income were also included in the questionnaire in order to assess their relationships to dementia literacy. Age demographics were stratified into young adults (18–39 years old), middle adulthood (40–59 years old), and older adults (60+ years old). Education was obtained by report of highest level of education completed and then categorized into post-secondary education (completion of bachelors or graduate degree), vocational education (completion of trade's apprenticeship or college diploma), and high school education (no post-secondary education completed of any kind). Income level categorization was determined by self-report as to whether individuals earned below, at, or above their country of residence's national average.

### Statistical Analysis

A two-tailed independent sample *t*-test bootstrapped at 1,000 permutations was conducted to examine sex differences in ratings of perception of dementia preventability. Additionally, one-way ANOVAs were conducted to evaluate the differences within age group, level of education, income, ethnicity, and country corrected for multiple comparisons using Tukey. To assess differences in risk scoring for the 11empirically supported risk factors, Kruskal-Wallis tests were conducted factored by demographic categorizations. Initial Kruskal-Wallis testing p-threshold was evaluated at *p* < 0.005 to account for repeated testing of the eleven empirically supported risk factors being evaluated. *Post-hoc* testing was also corrected for multiple comparisons using the same Bonferroni method as the omnibus testing. All analyses were conducted in SPSS Statistics (ver 24, 2019).

## Results

In total, 617 survey responses were collected which were further assessed for quality of responses. Six were excluded due to participants self-reporting at the end of the questionnaire that they did not feel they answered questions accurately. An additional five were excluded due to randomness of responses, verified by “failed catch trials” or incongruent responses and independently identified by the authors (B.R.H., J.A.F., and M.Y.S.W.). A further eight were excluded due to more than two demographic variables being unreported by the participant, as decided upon as exclusion criteria prior to data collection. Thus, a total of 598 responses were included in our analysis.

### Demographics

Demographic information for our sample is reported in Table 1. Of the total sample (*n* = 598), 388 respondents were classified as young adults, 149 as middle aged, 31 as older adults, and 30 declining to provide their age. The mean reported age was 39.2 (± 11.5) years old, and over half of our sample (60.7%, *n* = 362) reported their biological sex as male. Most of our sample was categorized as completing post-secondary education (81.4%, *n* = 487) or vocational education (4.7%, *n* = 28). Reported level of income indicated 29.4% (*n* = 176) reporting above average income, 44.6% (*n* = 267) at average income, and 22.9% (*n* = 137) reporting below average income. Reported ethnicity was 53.2% (*n* = 318) White and 45.7% (*n* = 268) Black, Indigenous or Person of Color (BIPOC). The sample was further characterized by 65.7% (*n* = 393) residing in the USA.

**Table 1:** Demographic characteristics of sample.

**Sample characteristics**	**Frequency (%)**
Age, mean (SD)	39.23 (12.2)
**Age group (years)**	
Young adults (18–39)	388 (64.9)
Middle aged (40–59)	149 (24.9)
Older adults (60+)	31 (5.2)
Not reported	30 (5.0)
**Sex**	
Male	362 (60.5)
Female	236 (39.5)
**Education**^a^	
High school education	82 (13.7)
Vocational education	28 (4.7)
Post-secondary education	487 (81.4)
Not reported	1 (.2)
**Income**	
Above national average	176 (29.4)
At national average	267 (44.6)
Below national average	137 (22.9)
Not reported	18 (3.0)
**Ethnicity**^b^	
White	318 (53.2)
BIPOC	268 (45.7)
Not reported	12 (2.0)
**Current Country of Residence**	
United states	393 (65.7)
India	175 (29.3)
Other^c^	30 (5.0)

a *Education was self-reported and categorized as follows: high school education (some high school, high school diploma), vocational education (trade school or college diploma) and post-secondary education (bachelor's or graduate degree).*

b *Ethnicity was self-reported and categorized as follows: White and Black, Indigenous or Person of Color (BIPOC).*

c *Other countries included: Brazil, Canada, France, Germany, Italy, Nigeria, Portugal, and Vietnam*.

### Belief of Dementia Prevention

**Figure 1:**
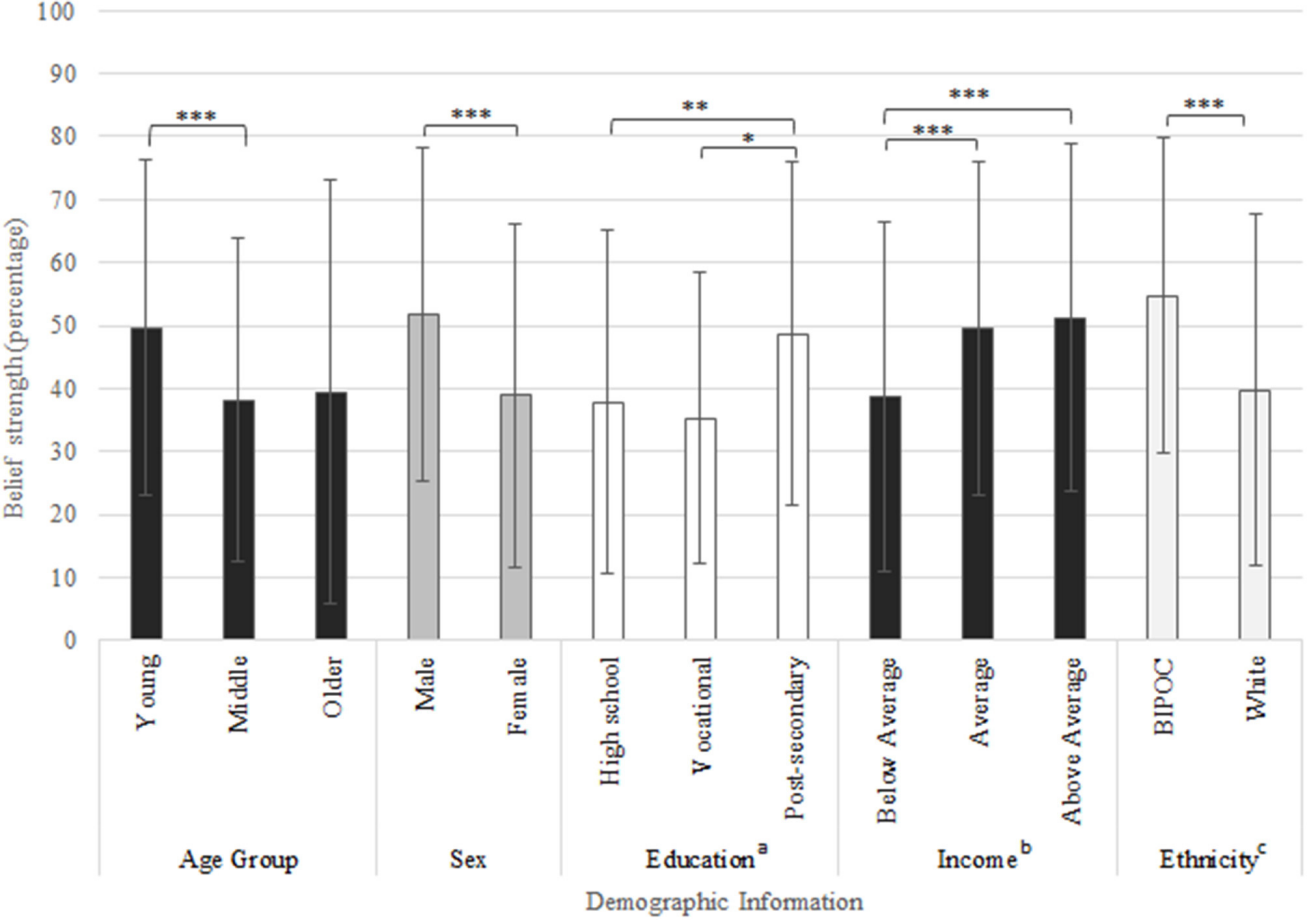
Belief ratings (0–100%) based on question “to what extent do you believe that Alzheimer's disease and dementia are preventable,” responses displayed by demographic subgroups. *Indicates *p*-value < 0.05, **indicates *p*-value < 0.01, ***indicates *p*-value < 0.001. Education was self-reported and categorized as follows: ^a^high school education (some high school, high school diploma), vocational education (trade school or college diploma) and post-secondary education (bachelor's or graduate degree). ^b^Ethnicity was self-reported and categorized as follows: White and Black, Indigenous or Person of Color (BIPOC). ^c^Other countries included: Brazil, Canada, France, Germany, Italy, Nigeria, Portugal, and Vietnam.

Almost all participants reported dementia as somewhat preventable, with average preventability score being 46.6% (±27.5%). Only 5% of the respondents indicated that dementia is completely unpreventable; comparably, 1.5% of the respondents indicated that they believed dementia is 100% preventable. Demographic subgroups demonstrated differences in their beliefs in the preventability of dementia (Figure 1). On average, women reported that dementia was less preventable compared to men (*t*_(596)_ = 5.676, *p* = 0.001). White individuals reported dementia as less preventable than BIPOC individuals (*t*_(584)_ = 6.841, *p* < 0.001). Omnibus testing for age (*F*_(2, 565)_ = 10.884, *p* < 0.001), education level (*F*_(2, 594)_ = 8.122, *p* < 0.001), income (*F*_(2, 577)_ = 11.120, *p* < 0.001), and country of residence (*F*_(2, 595)_ = 36.330, *p* < 0.001) all returned as significant. *Post-hoc* comparisons indicated younger adults (M = 49.6, SD = 26.6) believed dementia to be more preventable than middle aged adults (M = 38.15, SD = 25.7, *p* < 0.001). Respondents with post-secondary education (M = 48.7, SD = 27.4) believed dementia to be more preventable than both vocational education (M = 35.3, SD = 23.3, *p* = 0.030) and high school education (M = 37.9, SD = 27.1, *p* = 0.003). Those with below average income (M = 38.7, SD = 27.8) believed dementia was less preventable than what was reported by those with both average (M = 49.5, SD = 26.4, *p* < 0.001) and above average income (M = 51.2, SD = 27.5, *p* < 0.001). Respondents from India (M = 60.7, SD = 21.5) believed dementia to be more preventable than both residents of the USA (M = 41.0, SD = 27.4, *p* < 0.001) and other countries (M = 38.69, SD = 31.2, *p* < 0.001).

### General Population Dementia Risk Factor Beliefs

Of the entire sample, 63.9% were correctly able to identify all 11 empirically supported factors as having at least some risk for increasing dementia risk, while 9.1% were able to identify between one and five of the supported risk factors, and only 0.8% reported “no risk” for all 11 of the supported factors. Table 2 displays the relative frequencies of scoring for each of the 14 factors evaluated. Of the 11 supported risk factors, heart disease and smoking were the most frequently scored as having “no risk” (23.2 and 16.6%, respectively), while comparatively, old age and genetics were the least common to be reported as no risk (3.0 and 3.8%, respectively). The average score for each of the 11 supported risk factors is reported in **Table 4**. Notably, average risk score did not exceed 4.7, representing moderate risk, for any of these risk factors. Heart disease reported the lowest average score at 3.3, representing low risk.

**Table 2:** Frequency of risk scoring for the 14 potential risk factors for dementia.

**Risk factor**	**No risk (1)**		**Low risk (2–3)**		**Moderate risk (4–5)**		**Higher risk (6–7)**	
	**Frequency**	**%**	**Frequency**	**%**	**Frequency**	**%**	**Frequency**	**%**
Old age^*^	18	3.0	106	17.7	278	46.5	195	32.6
Genetics^*^	23	3.8	137	22.9	257	43.0	181	30.3
Exposure to toxins	41	6.9	196	32.8	254	42.5	107	17.9
Head injury^*^	45	7.5	185	30.9	242	40.5	125	20.9
Unhealthy diet^*^	56	9.4	171	28.6	269	45.0	96	16.1
Stress^*^	63	10.5	202	33.8	236	39.5	94	15.7
Physical inactivity^*^	76	12.7	178	29.8	245	41.0	97	16.2
Social isolation^*^	84	14.0	177	29.6	243	40.6	90	15.1
Alcohol^*^	87	14.5	191	31.9	225	37.6	91	15.2
Mental illness^*^	93	15.6	159	26.6	239	40.0	105	17.6
Smoking^*^	99	16.6	196	32.8	205	34.3	93	15.6
Virus or infection	115	19.2	183	30.6	212	35.5	86	14.4
Heart disease^*^	139	23.2	169	28.3	215	36.0	67	11.2
God's will	185	30.9	112	18.7	206	34.4	91	15.2

* *Indicates the eleven empirically supported factors for increased dementia risk*.

### Demographic Differences in Scoring of Risk Perception

After correction for multiple comparisons, all 11empirically supported risk factors demonstrated at least one relationship with a demographic characteristic (Table 3). Factors that were more frequently scored as low or no risk demonstrated more demographic relationships than those that were scored as high risk. Specific scoring differences for the 11 empirically supported factors are reported below by demographic category, with mean risk score reported in Table 4.

**Table 3:** Scoring distribution relationships with demographic variables using Kruskal-Wallis omnibus testing.

	**Age group**	**Sex**	**Education**	**Income**	**Ethnicity**	**Country**
Old age	–	–	–	–	O	–
Genetics	–	–	–	–	O	–
Head injury	–	O	–	–	O	–
Unhealthy diet	–	–	–	–	O	O
Stress	–	O	–	–	O	O
Physical inactivity	O	–	O	–	O	O
Social isolation	O	O	O	O	O	O
Alcohol	O	O	O	–	O	O
Mental illness	O	O	O	O	O	O
Smoking	O	O	O	O	O	O
Heart disease	O	O	O	O	O	O

*Indicates H statistic with p-value of <0.005*.

**Table 4:** Mean and standard deviations per demographic variable for risk score of the 11 empirically supported risk factor for increasing dementia risk.

		**Old age**		**Genetics**		**Head injury**		**Unhealthy**		**Stress**		**Physical**		**Social**		**Alcohol**		**Mental**		**Smoking**		**Heart**	
								**diet**				**inactivity**		**isolation**				**illness**				**disease**	
**Total sample mean score (SD)**		**4.7 (1.5)**		**4.5 (1.6)**		**4.0 (1.7)**		**3.9 (1.6)**		**3.7 (1.6)**		**3.7 (1.7)**		**3.7 (1.7)**		**3.6 (1.7)**		**3.7 (1.7)**		**3.5 (1.8)**		**3.3 (1.8)**	
		**M**	**SD**	**M**	**SD**	**M**	**SD**	**M**	**SD**	**M**	**SD**	**M**	**SD**	**M**	**SD**	**M**	**SD**	**M**	**SD**	**M**	**SD**	**M**	**SD**
Age group	Young	4.7	1.4	4.6	1.5	4.1	1.7	3.9	1.6	3.9	1.6	3.9^a^	1.6	3.8^a^	1.6	3.7^a^	1.6	3.9^a^	1.7	3.7^a^	1.7	3.5^a^	1.7
	Middle	4.7	1.6	4.5	1.8	3.9	1.7	3.6	1.7	3.4	1.6	3.3^a^	1.7	3.3^a^	1.8	3.2^a^	1.8	3.2^a^	1.9	3.1^a^	1.8	2.8^a^	1.8
	Older	4.8	1.7	4.6	1.8	4.2	2.0	4.2	1.9	3.3	1.8	3.7	1.9	3.5	1.7	3.5	2.1	3.3	2.0	3.1	1.8	3.1	1.8
Sex	Male	4.8	1.5	4.4	1.6	4.2^b^	1.7	3.9	1.5	3.9^b^	1.6	3.9	1.5	3.9^b^	1.7	3.8^b^	1.7	4.0^b^	1.7	3.8^b^	1.7	3.6^b^	1.7
	Female	4.6	1.6	4.7	1.6	3.8^b^	1.7	3.8	1.7	3.4^b^	1.7	3.6	1.7	3.4^b^	1.7	3.3^b^	1.7	3.3^b^	1.8	3.2^b^	1.8	2.9^b^	1.8
Education	High school	4.9	1.5	5.0	1.6	3.9	1.8	3.4	1.7	3.4	1.6	3.2^c^	1.7	3.1^c^	1.9	2.9^c^	1.7	3.1^c^	1.7	2.6^c^	1.6	2.5^c^	1.6
	Vocational	4.8	1.7	4.8	1.4	3.8	1.4	3.8	1.4	3.3	1.5	3.5	1.4	3.3	1.4	3.0	1.6	3.1	1.7	2.8	1.5	2.3^d^	1.5
	Post-secondary	4.7	1.5	4.5	1.6	4.1	1.7	4.0	1.6	3.8	1.6	3.9^c^	1.6	3.8^c^	1.7	3.7^c^	1.7	3.9^c^	1.8	3.7^c^	1.8	3.5^cd^	1.8
Income	Below average	4.7	1.5	4.7	1.7	3.9	1.7	3.7	1.5	3.5	1.6	3.5	1.5	3.4^f^	1.7	3.3	1.7	3.3^fh^	1.8	3.1^f^	1.7	2.9^f^	1.7
	Average	4.6	1.5	4.4	1.5	4.0	1.7	3.8	1.6	3.7	1.6	3.7	1.6	3.7	1.6	3.7	1.7	3.9^h^	1.7	3.6	1.8	3.3	1.8
	Above average	5.0	1.5	4.6	1.5	4.3	1.7	4.2	1.7	4.0	1.6	4.2	1.7	4.0^f^	1.7	3.8	1.7	3.9^f^	1.8	3.9^f^	1.8	3.8^f^	1.8
Ethnicity	BIPOC	4.5^i^	1.4	4.4^i^	1.5	4.3^i^	1.7	4.1^i^	1.5	4.1^i^	1.6	4.1^i^	1.5	4.0^i^	1.6	4.0^i^	1.5	4.3^i^	1.6	4.0^i^	1.7	3.9^i^	1.7
	White	4.9^i^	1.6	4.7^i^	1.7	3.8^i^	1.7	3.7^i^	1.7	3.4^i^	1.6	3.5^i^	1.7	3.4^i^	1.8	3.8^i^	1.6	3.3^i^	1.8	3.1^i^	1.7	2.8^i^	1.7
Country	USA	4.7	1.6	4.6	1.7	3.9	1.8	3.7^j^	1.7	3.5^j^	1.7	3.5^j^	1.7	3.5^j^	1.7	3.4^j^	1.7	3.4^j^	1.8	3.2^j^	1.7	3.0^j^	1.7
	India	4.6	1.2	4.4	1.3	4.3	1.6	4.3^jk^	1.3	4.3^j^	1.4	4.4^jk^	1.3	4.2^jk^	1.4	4.3^jk^	1.5	4.4^jk^	1.3	4.3^jk^	1.6	4.2^jk^	1.6
	Other	5.1	2.1	4.7	2.2	3.7	1.9	3.1^k^	1.8	3.6	2.0	3.1^k^	1.8	3.2^k^	1.9	2.8^k^	1.6	3.5^k^	2.1	2.9^k^	1.6	2.3^k^	1.6

a, b, c, d, e, f, g, h, I, j, k *Indicates significant differences in ordinal ranking of risk factor within demographic subgroup after post-hoc testing of Kruskal-Wallis omnibus and correcting for multiple comparisons*.

#### Age

Scoring for the risk factors of physical inactivity (H (2) = 13.890, *p* = 0.001), social isolation (H (2) = 11.476, *p* = 0.003), alcohol (H (2) = 11.949, *p* < 0.001), mental illness (H (2) = 20.587, *p* < 0.001), smoking (H (2) = 15.940, *p* < 0.001), and heart disease (H (2) = 17.744, *p* < 0.001) were significantly related with age. Significant rank differences were seen *post-hoc* between younger and middle aged adults for physical inactivity (*p* = 0.002), social isolation (*p* = 0.001), alcohol (*p* = 0.003), mental illness (*p* < 0.001), smoking (*p* < 0.001), and heart disease (*p* < 0.001), where the young adult group ranked these factors as higher risk for dementia. The remaining risk factors of old age, genetics, head injury, stress, and unhealthy diet were not observed to have a significant relationship with age after adjusting for multiple testing (*p* > 0.005).

#### Sex

Male respondents were found to indicate higher risk of dementia for head injury (H (1) = 8.196, *p* = 0.004), stress (H (1) = 15.442, *p* < 0.001), social isolation (H (1) = 9.687, *p* < 0.001), alcohol (H (1) = 12.269, *p* <0.001), mental illness (H (1) = 19.675, *p* < 0.001), smoking (H (1) = 16.229, *p* < 0.001), and heart disease (H (1) = 17.345, *p* < 0.001) compared to female respondents. The remaining supported risk factors of old age, genetics, unhealthy diet, and physical inactivity were not observed to have a significant relationship with sex after adjusting for multiple testing (*p* > 0.005).

#### Education

Scoring for the risk factors of physical inactivity (H(2) = 11.476, *p* = 0.003), social isolation (H (2) = 11.844, *p* = 0.003), alcohol (H(2) = 18.571, *p* < 0.001), mental illness (H (2) = 16.531, *p* = 0.001), smoking (H (2) = 31.028, *p* < 0.001), and heart disease (H (2) = 20.289, *p* < 0.001) were significantly related with education. Significant rank differences, after adjustment for multiple comparisons, were seen *post-hoc* between high school education and post-secondary education for physical inactivity (*p* = 0.003), social isolation (*p* = 0.004), mental illness (*p* = 0.001), smoking (*p* < 0.001), and heart disease (*p* < 0.001), as well as between high school education and vocational education groups for alcohol (*p* < 0.001) and smoking (*p* = 0.023). Specifically, those with high school education attainment ranked the above factors as having lower risk for dementia. Only heart disease was found *post-hoc* to have rank differences between vocational education and post-secondary education groups with vocational education scoring lower (*p* = 0.001). Old age, genetics, head injury, stress, and unhealthy diet were not significantly related to education (*p* > 0.005).

#### Income

Scoring for the risk factors of social isolation (H (2) = 12.200, *p* = 0.002), mental illness (H (2) = 11.528, *p* < 0.001), smoking (H (2) = 14.114, *p* < 0.001), and heart disease (H (2) = 20.289, *p* < 0.001) were observed to have a relationship with income. Those with lower income ranked the above factors as having lower risk for dementia in comparison to greater income earners. Significant rank differences were seen *post-hoc* between below average income and above average income for social isolation (*p* = 0.002), mental illness (*p* = 0.014), smoking (*p* = 0.001), and heart disease (*p* < 0.001), as well as, between below average income and average income for social isolation (*p* = 0.048), mental illness (*p* = 0.007), smoking (*p* = 0.035), and heart disease (*p* = 0.045). Further, in the *post-hoc* comparison for heart disease, average income earners reported lower risk scores compared to above average income earners (*p* = 0.045). Risk scores for old age, genetics, head injury, unhealthy diet, stress, physical inactivity, and alcohol were not significantly related to income after multiple testing corrections (*p* > 0.005).

#### Ethnicity

Risk rankings for all supported risk factors were observed to have a relationship with ethnicity. Head injury (H (1) = 8.070, *p* = 0.004), unhealthy diet (H (1) = 8.397, *p* = 0.004), stress (H (1) = 25.959, *p* < 0.001) physical inactivity (H (1) = 16.871, *p* < 0.001), social isolation (H (1) = 15.490, *p* < .001), alcohol (H (1) = 31.923, *p* < 0.001), mental illness (H (1) = 44.062, *p* < 0.001), smoking (H (1) = 29.832, *p* < 0.001), and heart disease (H (1) = 47.930, *p* < 0.001) were ranked as higher risk factors for dementia by BIPOC than Whites. Comparatively, old age (H (1) = 8.070, *p* = 0.004) and genetics were ranked as higher risk factors for dementia by Whites than BIPOC.

#### Country

Risk rankings for unhealthy diet (H (1) = 23.916, *p* < 0.001), stress (H (2) = 33.042, *p* < 0.001), physical inactivity (H (2) = 36.334, *p* <0.001), social isolation (H (2) = 27.848, *p* <0.001), alcohol (H (2) = 39.171, *p* < 0.001), mental illness (H (1) = 35.882, *p* < 0.001), smoking (H (2) = 46.294, *p* < 0.001), and heart disease (H (2) = 65.152, *p* < 0.001) were observed to have a relationship with country of residence. Follow-up *post-hoc* comparisons for the omnibus testing, after adjusting for multiple comparisons, showed that respondents from India ranked unhealthy diet, stress, physical inactivity, social isolation, smoking, mental illness, alcohol, and heart disease, with higher risk scores when compared to those from the United States (all comparisons *p* < 0.001). India compared to other countries indicated significantly higher risk scoring for social isolation (*p* = 0.004), mental illness (*p* = 0.020), alcohol (*p* < 0.001), heart disease (*p* < 0.001), physical inactivity, smoking, and unhealthy diet (*p* = 0.001). No significant differences were found for the comparison of the United States to other countries.

## Discussion

The aim of this study was to identify whether underlying demographic factors influenced dementia literacy and perceptions of risk factors related to dementia. Our survey captured the responses from 598 individuals of varying age, gender, education, income, ethnicity, and country of residence. Our results indicated that as an aggregated group the understanding that dementia is a preventable disease was high, but differences in the strength of this belief varied by demographic characteristics.

Overall, women reported lower belief ratings that dementia is a preventable disease compared to men. This result may be reflective of the historic narrative that women are at greater risk for dementia than men. However, this narrative has been challenged by large-cohort studies that have found no differences in the incidence of dementia before the age of 80 between sexes (14–16). Continued dissemination of information to the public that reflects dementia is a disease that affects both men and women is a priority. Furthermore, differences in risk scoring for modifiable lifestyle factors were found for sex. Men reported greater risk scores than women for the modifiable risk factors of head injury, social isolation, alcohol, mental illness, smoking, and heart disease, but no differences in risk scores were found for the other modifiable risk factors. It is unclear why these differences in perception in risk scoring may exist in the general population between sexes, as even within the scientific community little consensus has been drawn whether certain lifestyle factors carry more risk for a specific sex compared to another. This gap in the literature surrounding dementia risk is largely due to the fact that studies that examine risk factors for dementia often adjust for sex during analysis, even though many scenarios exist in where sex and gender differences may affect risk (17, 18). Arguably, the differences in risk perception within our sample may be reflective of higher educational attainment of the men in the sample. However, this interaction was not explored within our analysis, nor can higher education be definitively confirmed as the reason for scoring differences, though it may be a contributing factor.

Our study also found that, in regard to specific factors related to increasing dementia risk, most of the sample was accurately able to recognize multiple empirically supported factors as having some contribution toward increasing dementia risk. This finding is in line with a previous review in which results suggested that over the past decade, the general public has increasingly become more informed and aware of risk factors related to increasing dementia risk (2, 13). In our sample specifically, old age and genetics were risk factors that the sample most frequently scored as having some associated risk for increasing dementia risk. Comparatively, associated risk scores were generally scored low indicating room for improvement, especially regarding perceptions of modifiable lifestyle risk factors.

Of the modifiable risk factors we included in our survey, heart disease was the most frequently scored factor as having no risk for increasing dementia, with 23.2% of the sample selecting the no risk scoring option and an additional 28.3% scoring heart disease as low risk. Smoking was similarly scored, with 16.6% of the sample selecting no risk for increasing dementia risk and 32.8% scoring smoking as low risk. Critically, both heart disease and smoking have increasingly been shown to be modifiable lifestyle factors responsible for increasing dementia risk (19, 20). Yet, there appears to be a disconnect between the public perception of heart disease and smoking as risk factors for dementia and what has been empirically supported. A similar disconnected relationship between perception of risk and empirical evidence existed in our sample for the other modifiable risk factors, where 38–46% of the sample used the no risk or low risk scoring option, depending on the risk factor being evaluated. Continued education surrounding the risks of modifiable lifestyle factors related to dementia should continue to be a priority for the general population. Additionally, further investigation into how various beliefs compare to specific metrics of population attributable risk or hazard ratios could provide more targeted evidence toward which factors to address for specific demographic characteristics. Notably, this is an important facet to consider since different risk factors will have various levels of influence on individual risk vs. a population-wide effect.

Our results indicate that certain demographic characteristics influence the scoring of how much risk individuals associate with modifiable risk factors. We found that younger adults scored the empirically supported modifiable risk factors, such as physical inactivity, social isolation, alcohol, mental illness, smoking, and heart disease, as higher risk for dementia compared to the older adult age groups. The fact that younger adults recognize these modifiable factors as higher risk is advantageous from a public health perspective, as implementation of lifestyle changes to decrease dementia risk should occur as early as possible. Dementia education targeting young and middle-aged adults should be prioritized for the prevention of dementia, as it is becoming increasingly recognized that the disease often starts 10 years before the onset of clinical symptoms (21).

Examining the scoring differences within education as well as income demographics indicated that varying levels of educational attainment had differing risk scoring for several modifiable risk factors. These differences mainly occurred between those with high school education and those with post-secondary education. However, for the overall lowest scored risk factors of heart disease and smoking differences in scoring were observed between all three groups. Similar relationships were also observed for level of income. Understandably from a socioeconomic perspective, individuals with higher levels of educational attainment and income tend to have access to resources and materials that may influence how they perceive risk factors associated with dementia. Nevertheless, it should be noted that within our sample even those who reported higher levels of educational attainment and income still scored many of the modifiable risk factors in the low to moderate range, contrary to level of risk that has been reported in empirical studies (6). Therefore, increasing risk perception awareness of modifiable risk factors appears to be a need for all levels of education and income, but specific attention should continue to be focused on individuals with lower incomes and education.

When comparing ethnicities, BIPOC participants scored all modifiable risk factors higher than White participants. Additionally, BIPOC participants also reported greater preventability beliefs than Whites, potentially indicating that there is an understanding of the link between modifiable risk factors and dementia preventability (22). Furthermore, country of residence demonstrated differences in risk scoring for modifiable lifestyle factors, except for head injury. Dementia continues to be a global issue, yet the urgency and prioritization of dementia education varies between countries (23). As the global prevalence of dementia continues to grow, public health education targeted toward modifiable risk factors should continue to be a priority, especially for low-to-middle-income countries where both life expectancy and cardiovascular risk factors are increasing rapidly (24).

Our study, although large in scale and including global responses, is not without limitations. First of which, our sample consisted mainly of White, American individuals who had high levels of educational attainment. A larger data set may be useful in capturing a larger representation of ethnicities and countries of residence that tend to have limited evaluations in regard to dementia perceptions (2). Furthermore, the respondent pool may also be limited to those who read and comprehend English, and those who are comfortable using computer technologies and the mTurk platform. However, evidence has shown that responses via mTurk are comparable to those done in a lab not via computer interface (25). Additionally, the demographic characteristics evaluated within this study were done so in isolation from each other. The interactions between these demographics and how they are interrelated should also be considered, especially when creating and implementing public health education strategies.

The results of our survey indicate that various demographic characteristics, such as, sex, age cohort, education, income, ethnicity and country of residence are related to how individuals perceive dementia as a preventable disease and how individuals perceive associated risk to various established risk factors of dementia. While specific demographic factors have been identified related to risk perception, it is also clear that, regardless of demographic variables, modifiable risk factors are areas that need continued education. Public health education and initiatives for dementia prevention should focus on modifiable risk factors, especially cardiovascular related elements, in addition to considering the barriers related to demographic factors that may prevent populations from accessing programs and information. Future research should investigate the relationship between risk factor literacy, participation in risk reducing behavior, and perceived barriers to participating in risk reducing behaviors.

## Data Availability Statement

The raw data supporting the conclusions of this article will be made available by the authors, without undue reservation.

## Ethics Statement

The studies involving human participants were reviewed and approved by Western Research Ethics Board—Non-Medical Research Ethics Board. The participants provided their written informed consent to participate in this study.

## Author Contributions

LN conceived the study and was in charge of overall direction and planning. BRH was the lead on importing survey questions to Qualtrics, creating mTurk work task, analyzed the data with input from LN, and wrote the manuscript with input from all authors. All authors collaborated in the design and creation of survey content.

## Conflict of Interest

The authors declare that the research was conducted in the absence of any commercial or financial relationships that could be construed as a potential conflict of interest.
